# 3-(2,5-Dimethyl­furan-3-yl)-1*H*-pyrazol-5-ol–ethyl 3-(propan-2-yl­idene)carbazate (1/1)

**DOI:** 10.1107/S1600536810043886

**Published:** 2010-10-31

**Authors:** Tara Shahani, Hoong-Kun Fun, R. Venkat Ragavan, V. Vijayakumar, S. Sarveswari

**Affiliations:** a X-ray Crystallography Unit, School of Physics, Universiti Sains Malaysia, 11800 USM, Penang, Malaysia; b Department of Studies in Chemistry, Mangalore University, Mangalagangotri, Mangalore 574 199, India

## Abstract

In the title 1:1 adduct, C_6_H_12_N_2_O_2_·C_9_H_10_N_2_O_2_, the maximum deviations from the 1*H*-pyrazole-5-ol and furan rings are 0.014 (1) and 0.003 (1) Å, respectively. The dihedral angle formed between the 1*H*-pyrazol-5-ol and 2,5-dimethyl­furan rings is 21.07 (5)°. In the crystal, pairs of inter­molecular O—H⋯N hydrogen bonds form inversion dimers of the 3-(2,5-dimethyl­furan-3-yl)-1*H*-pyrazol-5-ol species, generating *R* ^2^_2_(8) ring motifs. Mol­ecules are further linked by inter­molecular N—H⋯O, N—H⋯N and C—H⋯O hydrogen bonds to form ribbons along the [010] direction containing bifurcated *R*_1_^2^(5) and *R*_2_^1^(7) ring motifs. Further stablization of the packing is provided by weak π–π [centroid–centroid distance = 3.5686 (15) Å] and C—H⋯π inter­actions.

## Related literature

For pyrazole derivatives and their microbial activities, see: Ragavan *et al.* (2009 ▶, 2010 ▶). For a related structure, see: Shahani *et al.* (2010 ▶). For ring conformations, see: Cremer & Pople (1975 ▶). For hydrogen-bond motifs, see: Bernstein *et al.* (1995 ▶). For bond-length data, see: Allen *et al.* (1987 ▶). For the stability of the temperature controller used for the data collection, see: Cosier & Glazer (1986 ▶).
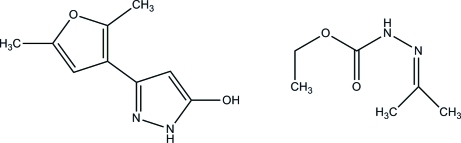


## Experimental

### 

#### Crystal data

C_6_H_12_N_2_O_2_·C_9_H_10_N_2_O_2_
*M* *_r_* = 322.37
Triclinic, 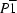

*a* = 8.6988 (17) Å
*b* = 9.4830 (19) Å
*c* = 11.837 (4) Å
α = 107.293 (4)°
β = 100.354 (5)°
γ = 108.346 (3)°
*V* = 843.7 (3) Å^3^
*Z* = 2
Mo *K*α radiation
μ = 0.09 mm^−1^
*T* = 100 K
0.40 × 0.21 × 0.13 mm

#### Data collection

Bruker APEXII DUO CCD diffractometer
Absorption correction: multi-scan (*SADABS*; Bruker, 2009 ▶) *T*_min_ = 0.964, *T*_max_ = 0.988
13322 measured reflections
3264 independent reflections
2911 reflections with *I* > 2σ(*I*)
*R*_int_ = 0.026

#### Refinement

*R*[*F* ^2^ > 2σ(*F* ^2^)] = 0.033
*wR*(*F* ^2^) = 0.099
*S* = 1.11
3264 reflections
225 parameters
H atoms treated by a mixture of independent and constrained refinement
Δρ_max_ = 0.29 e Å^−3^
Δρ_min_ = −0.22 e Å^−3^

### 

Data collection: *APEX2* (Bruker, 2009 ▶); cell refinement: *SAINT* (Bruker, 2009 ▶); data reduction: *SAINT*; program(s) used to solve structure: *SHELXTL* (Sheldrick, 2008 ▶); program(s) used to refine structure: *SHELXTL*; molecular graphics: *SHELXTL*; software used to prepare material for publication: *SHELXTL* and *PLATON* (Spek, 2009 ▶).

## Supplementary Material

Crystal structure: contains datablocks global, I. DOI: 10.1107/S1600536810043886/hb5696sup1.cif

Structure factors: contains datablocks I. DOI: 10.1107/S1600536810043886/hb5696Isup2.hkl

Additional supplementary materials:  crystallographic information; 3D view; checkCIF report

## Figures and Tables

**Table 1:** Hydrogen-bond geometry (Å, °)

*D*—H⋯*A*	*D*—H	H⋯*A*	*D*⋯*A*	*D*—H⋯*A*
N1—H1*N*1⋯O4^i^	0.883 (18)	2.304 (19)	3.0363 (19)	140.1 (16)
N1—H1*N*1⋯N4^i^	0.883 (18)	2.288 (19)	3.043 (2)	143.1 (16)
N3—H1*N*3⋯O2^ii^	0.872 (18)	2.076 (19)	2.9293 (19)	166.7 (18)
O2—H1*O*2⋯N2^iii^	0.93 (2)	1.73 (2)	2.6602 (17)	176 (2)
C5—H5*A*⋯O4^i^	0.93	2.35	3.212 (2)	153
C11—H11*B*⋯*Cg*2^iv^	0.97	2.71	3.50 (2)	138

Symmetry codes: (i) 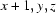; (ii) 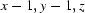; (iii) 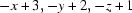; (iv) 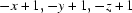. *Cg*2 is the centroid of the 1*H*-pyrazole ring (N1/N2/C1–C3).

**Fig. 1.**
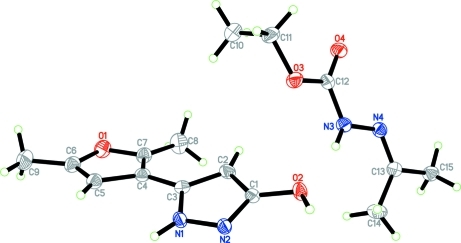
The molecular structure of the title compound, showing 50% probability displacement ellipsoids.

**Fig. 2.**
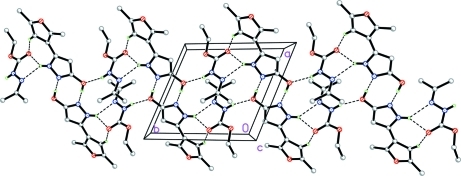
The crystal packing of the title compound, viewed along c axis, showing ribbons along the [010] direction..

## supplementary crystallographic information

### Comment

Antibacterial and antifungal activities of the azoles are most widely studied and some of them are in clinical practice as anti-microbial agents. However, the azole-resistant strain had led to the development of new antimicrobial compounds. In particular, pyrazole derivatives are extensively studied and used as antimicrobial agents. Pyrazole is an important class of heterocyclic compounds and many pyrazole derivatives are reported to have the broad spectrum of biological properties such as anti-inflammatory, antifungal, herbicidal, anti-tumour, cytotoxic, molecular modelling and antiviral activities. Pyrazole derivatives also act as antiangiogenic agents, A3 adenosine receptor antagonists, neuropeptide YY5 receptor antagonists, kinase inhibitor for treatment of type 2 diabetes, hyperlipidemia, obesity, and thrombopiotinmimetics. Recently urea derivatives of pyrazoles have been reported as potent inhibitors of p38 kinase. Since the high electronegativity of halogens (particularly chlorine and fluorine) in the aromatic part of the drug molecules play an important role in enhancing their biological activity, we are interested to have 4-fluoro or 4-chloro substitution in the aryls of 1,5-diaryl pyrazoles. As part of our on-going research aiming the synthesis of new antimicrobial compounds, we have reported the synthesis of novel pyrazole derivatives and their microbial activities (Ragavan *et al.*, 2009; 2010). The structure of the title compound is presented here.

The asymmetric unit of the title compound, (Fig. 1), consists of one 3-(2,5-dimethylfuran-3-yl)-1*H*-pyrazol-5-ol and one ethyl2- (propan-2-ylidene)hydrazine carboxylate. The maximum deviations in 1*H*- pyrazole-5-ol (N1/N2/C1–C3/O2) and furan (C4–C7/O1) rings are 0.014 (1) and 0.003 (1) Å at atoms C2 and C7, respectively. The dihedral angles formed between the 1*H*-pyrazole-5-ol ring and 2,5-dimethylfuran ring (C4–C9/O1) is 21.07 (5)°. The bond lengths (Allen *et al.*,1987) and angles are within normal ranges and comparable to those closely related structures (Shahani *et al.*, 2010).

In the crystal packing (Fig. 2), pairs of intermolecular N2—H1O2···O2 hydrogen bonds form dimers with neighbouring molecules, generating *R*^2^_2_(8) ring motif. Furthermore N1—H1N1···O4, N1—H1N1···N4, N3—H1N3···O2, O2—H1O2···N2 and C5—H5A···O4 hydrogen bonds (Table 1) link the molecules into ribbons along [010] direction with bifurcated *R*_1_^2^(5) and *R*_2_^1^(7) ring motifs. The crystal structure is stablilized by weak π–π and C—H···π interactions.interactions [cg1···cg1=3.5686 (15) Å, symmetry code, 2-*X*, 1-Y, –*Z*], cg1 is the centroids of the 1*H*-pyrazole ring (N1/N2/C1—C3) and cg2 is the centroids of the 1*H*-pyrazole ring (N1/N2/C1–C3).

### Experimental

The compound has been synthesized using the method available in the literature (Ragavan *et al.*,2009) and recrystallized using the ethanol-chloroform 1:1 mixture to yield colourless blocks of (I). Yield: 78%. *M*.pt: 225.5–227.5 °C.

### Refinement

The hydrogen atoms bound to C atoms were positioned geometrically [C–H = 0.96–0.97 Å] with *U*_iso_(H) =1.2 or 1.5*U*_iso_(C). A rotating group model was applied to the methyl groups. The hydrogen atoms attached to the N and O atoms were located from the difference map and refined freely, [N–H = 0.872 (18)–0.883 (18) Å, O–H = 0.93 Å].

### Crystal data

**Table:**

C_6_H_12_N_2_O_2_·C_9_H_10_N_2_O_2_	*Z* = 2
*M**_r_* = 322.37	*F*(000) = 344
Triclinic, *P*1	*D*_x_ = 1.269 Mg m^−^^3^
Hall symbol: -P 1	Mo *K*α radiation, λ = 0.71073 Å
*a* = 8.6988 (17) Å	Cell parameters from 7782 reflections
*b* = 9.4830 (19) Å	θ = 2.6–31.7°
*c* = 11.837 (4) Å	µ = 0.09 mm^−^^1^
α = 107.293 (4)°	*T* = 100 K
β = 100.354 (5)°	Block, colourless
γ = 108.346 (3)°	0.40 × 0.21 × 0.13 mm
*V* = 843.7 (3) Å^3^	

### Data collection

**Table:**

Bruker APEXII DUO CCD diffractometer	3264 independent reflections
Radiation source: fine-focus sealed tube	2911 reflections with *I* > 2σ(*I*)
graphite	*R*_int_ = 0.026
φ and ω scans	θ_max_ = 26.0°, θ_min_ = 1.9°
Absorption correction: multi-scan (*SADABS*; Bruker, 2009)	*h* = −10→10
*T*_min_ = 0.964, *T*_max_ = 0.988	*k* = −11→11
13322 measured reflections	*l* = −14→14

### Refinement

**Table:**

Refinement on *F*^2^	Primary atom site location: structure-invariant direct methods
Least-squares matrix: full	Secondary atom site location: difference Fourier map
*R*[*F*^2^ > 2σ(*F*^2^)] = 0.033	Hydrogen site location: inferred from neighbouring sites
*wR*(*F*^2^) = 0.099	H atoms treated by a mixture of independent and constrained refinement
*S* = 1.11	*w* = 1/[σ^2^(*F*_o_^2^) + (0.045*P*)^2^ + 0.2939*P*] where *P* = (*F*_o_^2^ + 2*F*_c_^2^)/3
3264 reflections	(Δ/σ)_max_ < 0.001
225 parameters	Δρ_max_ = 0.28 e Å^−^^3^
0 restraints	Δρ_min_ = −0.22 e Å^−^^3^

### Special details

**Table:**

Experimental. The crystal was placed in the cold stream of an Oxford Cyrosystems Cobra open-flow nitrogen cryostat (Cosier & Glazer, 1986) operating at 100.0 (1) K.
Geometry. All e.s.d.'s (except the e.s.d. in the dihedral angle between two l.s. planes) are estimated using the full covariance matrix. The cell e.s.d.'s are taken into account individually in the estimation of e.s.d.'s in distances, angles and torsion angles; correlations between e.s.d.'s in cell parameters are only used when they are defined by crystal symmetry. An approximate (isotropic) treatment of cell e.s.d.'s is used for estimating e.s.d.'s involving l.s. planes.
Refinement. Refinement of *F*^2^ against ALL reflections. The weighted *R*-factor *wR* and goodness of fit *S* are based on *F*^2^, conventional *R*-factors *R* are based on *F*, with *F* set to zero for negative *F*^2^. The threshold expression of *F*^2^ > σ(*F*^2^) is used only for calculating *R*-factors(gt) *etc*. and is not relevant to the choice of reflections for refinement. *R*-factors based on *F*^2^ are statistically about twice as large as those based on *F*, and *R*- factors based on ALL data will be even larger.

### Fractional atomic coordinates and isotropic or equivalent isotropic displacement parameters (Å^2^)

**Table:**

	*x*	*y*	*z*	*U*_iso_*/*U*_eq_	
O1	0.72298 (12)	0.49965 (12)	−0.03577 (8)	0.0272 (2)	
O2	1.41202 (12)	1.13027 (10)	0.43430 (9)	0.0232 (2)	
N1	1.21447 (13)	0.71967 (13)	0.29969 (10)	0.0197 (2)	
N2	1.34466 (13)	0.85605 (12)	0.38574 (9)	0.0190 (2)	
C1	1.31074 (15)	0.97528 (15)	0.36573 (11)	0.0185 (3)	
C2	1.16205 (16)	0.91874 (15)	0.26795 (12)	0.0211 (3)	
H2A	1.1136	0.9791	0.2366	0.025*	
C3	1.10286 (15)	0.75316 (15)	0.22806 (11)	0.0185 (3)	
C4	0.95185 (16)	0.62751 (15)	0.13126 (11)	0.0201 (3)	
C5	0.87164 (16)	0.46384 (15)	0.11997 (12)	0.0222 (3)	
H5A	0.9076	0.4169	0.1725	0.027*	
C6	0.73478 (17)	0.39202 (16)	0.01874 (12)	0.0252 (3)	
C7	0.85745 (16)	0.64286 (16)	0.03429 (12)	0.0240 (3)	
C8	0.8742 (2)	0.77483 (18)	−0.01199 (14)	0.0344 (3)	
H8A	0.9922	0.8414	0.0089	0.052*	
H8B	0.8166	0.8382	0.0257	0.052*	
H8C	0.8246	0.7301	−0.1005	0.052*	
C9	0.59939 (19)	0.22804 (18)	−0.04258 (14)	0.0342 (3)	
H9A	0.6186	0.1632	0.0027	0.051*	
H9B	0.6011	0.1810	−0.1260	0.051*	
H9C	0.4909	0.2338	−0.0441	0.051*	
O3	0.08857 (11)	0.22838 (11)	0.42456 (9)	0.0236 (2)	
O4	0.06433 (11)	0.42612 (11)	0.36063 (9)	0.0244 (2)	
N3	0.29597 (14)	0.35988 (13)	0.36500 (10)	0.0209 (2)	
N4	0.37772 (13)	0.48909 (12)	0.33504 (10)	0.0201 (2)	
C10	−0.21443 (18)	0.07821 (18)	0.31978 (15)	0.0346 (3)	
H10A	−0.3233	0.0471	0.3338	0.052*	
H10B	−0.1904	−0.0153	0.2871	0.052*	
H10C	−0.2154	0.1302	0.2615	0.052*	
C11	−0.07996 (17)	0.19197 (17)	0.44018 (13)	0.0267 (3)	
H11A	−0.0984	0.2905	0.4695	0.032*	
H11B	−0.0878	0.1443	0.5021	0.032*	
C12	0.14196 (16)	0.34622 (14)	0.38212 (11)	0.0194 (3)	
C13	0.52197 (16)	0.50193 (15)	0.31731 (11)	0.0212 (3)	
C14	0.60676 (18)	0.38865 (18)	0.32513 (14)	0.0300 (3)	
H14A	0.6155	0.3777	0.4039	0.045*	
H14B	0.7183	0.4299	0.3169	0.045*	
H14C	0.5408	0.2856	0.2596	0.045*	
C15	0.61073 (17)	0.64131 (16)	0.28562 (13)	0.0249 (3)	
H15A	0.5436	0.7044	0.2843	0.037*	
H15B	0.6259	0.6025	0.2054	0.037*	
H15C	0.7196	0.7063	0.3468	0.037*	
H1N1	1.211 (2)	0.626 (2)	0.3012 (15)	0.033 (4)*	
H1N3	0.347 (2)	0.304 (2)	0.3897 (15)	0.031 (4)*	
H1O2	1.496 (3)	1.138 (2)	0.500 (2)	0.057 (6)*	

### Atomic displacement parameters (Å^2^)

**Table:**

	*U*^11^	*U*^22^	*U*^33^	*U*^12^	*U*^13^	*U*^23^
O1	0.0228 (5)	0.0284 (5)	0.0227 (5)	0.0062 (4)	0.0009 (4)	0.0068 (4)
O2	0.0222 (5)	0.0154 (4)	0.0274 (5)	0.0068 (4)	−0.0018 (4)	0.0078 (4)
N1	0.0200 (5)	0.0146 (5)	0.0221 (5)	0.0065 (4)	0.0026 (4)	0.0061 (4)
N2	0.0178 (5)	0.0157 (5)	0.0215 (5)	0.0065 (4)	0.0028 (4)	0.0061 (4)
C1	0.0190 (6)	0.0173 (6)	0.0212 (6)	0.0085 (5)	0.0067 (5)	0.0080 (5)
C2	0.0209 (6)	0.0212 (6)	0.0231 (6)	0.0115 (5)	0.0041 (5)	0.0090 (5)
C3	0.0174 (6)	0.0216 (6)	0.0187 (6)	0.0095 (5)	0.0070 (5)	0.0079 (5)
C4	0.0190 (6)	0.0218 (6)	0.0194 (6)	0.0090 (5)	0.0073 (5)	0.0059 (5)
C5	0.0215 (6)	0.0224 (6)	0.0217 (6)	0.0084 (5)	0.0074 (5)	0.0069 (5)
C6	0.0233 (7)	0.0258 (7)	0.0243 (7)	0.0080 (5)	0.0089 (5)	0.0073 (5)
C7	0.0211 (6)	0.0246 (7)	0.0221 (6)	0.0075 (5)	0.0043 (5)	0.0060 (5)
C8	0.0364 (8)	0.0329 (8)	0.0294 (7)	0.0115 (7)	−0.0003 (6)	0.0139 (6)
C9	0.0279 (7)	0.0295 (8)	0.0323 (8)	0.0019 (6)	0.0049 (6)	0.0067 (6)
O3	0.0212 (5)	0.0240 (5)	0.0319 (5)	0.0101 (4)	0.0109 (4)	0.0159 (4)
O4	0.0234 (5)	0.0235 (5)	0.0314 (5)	0.0129 (4)	0.0088 (4)	0.0133 (4)
N3	0.0214 (5)	0.0195 (5)	0.0282 (6)	0.0108 (5)	0.0090 (4)	0.0139 (5)
N4	0.0217 (5)	0.0185 (5)	0.0218 (5)	0.0084 (4)	0.0064 (4)	0.0093 (4)
C10	0.0259 (7)	0.0307 (8)	0.0430 (9)	0.0076 (6)	0.0118 (6)	0.0112 (7)
C11	0.0232 (7)	0.0288 (7)	0.0340 (7)	0.0104 (6)	0.0152 (6)	0.0156 (6)
C12	0.0218 (6)	0.0168 (6)	0.0187 (6)	0.0079 (5)	0.0045 (5)	0.0063 (5)
C13	0.0221 (6)	0.0226 (6)	0.0196 (6)	0.0100 (5)	0.0061 (5)	0.0079 (5)
C14	0.0310 (7)	0.0334 (8)	0.0411 (8)	0.0207 (6)	0.0195 (6)	0.0215 (7)
C15	0.0234 (7)	0.0261 (7)	0.0293 (7)	0.0108 (5)	0.0100 (5)	0.0138 (6)

### Geometric parameters (Å, °)

**Table:**

O1—C7	1.3719 (16)	C9—H9C	0.9600
O1—C6	1.3792 (17)	O3—C12	1.3445 (15)
O2—C1	1.3468 (15)	O3—C11	1.4558 (15)
O2—H1O2	0.93 (2)	O4—C12	1.2124 (15)
N1—C3	1.3493 (16)	N3—C12	1.3625 (17)
N1—N2	1.3685 (14)	N3—N4	1.3905 (15)
N1—H1N1	0.883 (18)	N3—H1N3	0.872 (18)
N2—C1	1.3288 (16)	N4—C13	1.2834 (17)
C1—C2	1.3988 (18)	C10—C11	1.502 (2)
C2—C3	1.3844 (18)	C10—H10A	0.9600
C2—H2A	0.9300	C10—H10B	0.9600
C3—C4	1.4561 (18)	C10—H10C	0.9600
C4—C7	1.3597 (19)	C11—H11A	0.9700
C4—C5	1.4405 (18)	C11—H11B	0.9700
C5—C6	1.3448 (19)	C13—C14	1.4967 (19)
C5—H5A	0.9300	C13—C15	1.4967 (18)
C6—C9	1.4835 (19)	C14—H14A	0.9600
C7—C8	1.486 (2)	C14—H14B	0.9600
C8—H8A	0.9600	C14—H14C	0.9600
C8—H8B	0.9600	C15—H15A	0.9600
C8—H8C	0.9600	C15—H15B	0.9600
C9—H9A	0.9600	C15—H15C	0.9600
C9—H9B	0.9600		
			
C7—O1—C6	107.17 (10)	H9A—C9—H9C	109.5
C1—O2—H1O2	110.5 (13)	H9B—C9—H9C	109.5
C3—N1—N2	111.95 (10)	C12—O3—C11	115.88 (10)
C3—N1—H1N1	129.8 (11)	C12—N3—N4	116.20 (11)
N2—N1—H1N1	118.1 (11)	C12—N3—H1N3	119.0 (11)
C1—N2—N1	104.49 (10)	N4—N3—H1N3	123.2 (11)
N2—C1—O2	121.83 (11)	C13—N4—N3	116.67 (11)
N2—C1—C2	111.95 (11)	C11—C10—H10A	109.5
O2—C1—C2	126.22 (11)	C11—C10—H10B	109.5
C3—C2—C1	104.84 (11)	H10A—C10—H10B	109.5
C3—C2—H2A	127.6	C11—C10—H10C	109.5
C1—C2—H2A	127.6	H10A—C10—H10C	109.5
N1—C3—C2	106.77 (11)	H10B—C10—H10C	109.5
N1—C3—C4	122.08 (11)	O3—C11—C10	110.77 (11)
C2—C3—C4	131.15 (12)	O3—C11—H11A	109.5
C7—C4—C5	106.49 (11)	C10—C11—H11A	109.5
C7—C4—C3	126.43 (12)	O3—C11—H11B	109.5
C5—C4—C3	127.08 (12)	C10—C11—H11B	109.5
C6—C5—C4	106.74 (12)	H11A—C11—H11B	108.1
C6—C5—H5A	126.6	O4—C12—O3	125.45 (12)
C4—C5—H5A	126.6	O4—C12—N3	125.31 (12)
C5—C6—O1	109.97 (12)	O3—C12—N3	109.22 (10)
C5—C6—C9	134.00 (13)	N4—C13—C14	125.22 (12)
O1—C6—C9	116.02 (12)	N4—C13—C15	116.82 (12)
C4—C7—O1	109.62 (12)	C14—C13—C15	117.96 (11)
C4—C7—C8	134.17 (13)	C13—C14—H14A	109.5
O1—C7—C8	116.15 (11)	C13—C14—H14B	109.5
C7—C8—H8A	109.5	H14A—C14—H14B	109.5
C7—C8—H8B	109.5	C13—C14—H14C	109.5
H8A—C8—H8B	109.5	H14A—C14—H14C	109.5
C7—C8—H8C	109.5	H14B—C14—H14C	109.5
H8A—C8—H8C	109.5	C13—C15—H15A	109.5
H8B—C8—H8C	109.5	C13—C15—H15B	109.5
C6—C9—H9A	109.5	H15A—C15—H15B	109.5
C6—C9—H9B	109.5	C13—C15—H15C	109.5
H9A—C9—H9B	109.5	H15A—C15—H15C	109.5
C6—C9—H9C	109.5	H15B—C15—H15C	109.5
			
C3—N1—N2—C1	0.09 (13)	C7—O1—C6—C5	0.06 (14)
N1—N2—C1—O2	179.58 (11)	C7—O1—C6—C9	179.60 (11)
N1—N2—C1—C2	−0.44 (14)	C5—C4—C7—O1	0.55 (14)
N2—C1—C2—C3	0.62 (14)	C3—C4—C7—O1	−178.68 (11)
O2—C1—C2—C3	−179.40 (11)	C5—C4—C7—C8	−176.26 (16)
N2—N1—C3—C2	0.30 (14)	C3—C4—C7—C8	4.5 (2)
N2—N1—C3—C4	−178.86 (10)	C6—O1—C7—C4	−0.39 (14)
C1—C2—C3—N1	−0.53 (13)	C6—O1—C7—C8	177.06 (12)
C1—C2—C3—C4	178.51 (12)	C12—N3—N4—C13	−179.21 (11)
N1—C3—C4—C7	−161.23 (13)	C12—O3—C11—C10	84.10 (14)
C2—C3—C4—C7	19.8 (2)	C11—O3—C12—O4	2.70 (18)
N1—C3—C4—C5	19.70 (19)	C11—O3—C12—N3	−176.27 (10)
C2—C3—C4—C5	−159.23 (13)	N4—N3—C12—O4	8.26 (18)
C7—C4—C5—C6	−0.50 (14)	N4—N3—C12—O3	−172.77 (10)
C3—C4—C5—C6	178.72 (12)	N3—N4—C13—C14	0.65 (19)
C4—C5—C6—O1	0.27 (14)	N3—N4—C13—C15	−179.72 (11)
C4—C5—C6—C9	−179.15 (15)		

### Hydrogen-bond geometry (Å, °)

**Table:**

*D*—H···*A*	*D*—H	H···*A*	*D*···*A*	*D*—H···*A*
N1—H1N1···O4^i^	0.883 (18)	2.304 (19)	3.0363 (19)	140.1 (16)
N1—H1N1···N4^i^	0.883 (18)	2.288 (19)	3.043 (2)	143.1 (16)
N3—H1N3···O2^ii^	0.872 (18)	2.076 (19)	2.9293 (19)	166.7 (18)
O2—H1O2···N2^iii^	0.93 (2)	1.73 (2)	2.6602 (17)	176 (2)
C5—H5A···O4^i^	0.93	2.35	3.212 (2)	153
C11—H11B···Cg2^iv^	0.97	2.71	3.498 (20)	138

Symmetry codes: (i) *x*+1, *y*, *z*; (ii) *x*−1, *y*−1, *z*; (iii) −*x*+3, −*y*+2, −*z*+1; (iv) −*x*+1, −*y*+1, −*z*+1.
