# 3-(2,5-Dimethyl­furan-3-yl)-1*H*-pyrazol-5-ol–ethyl 3-(propan-2-yl­idene)carbazate (1/1). Corrigendum

**DOI:** 10.1107/S1600536810047859

**Published:** 2010-11-23

**Authors:** Tara Shahani, Hoong-Kun Fun, R. Venkat Ragavan, V. Vijayakumar, S. Sarveswari

**Affiliations:** a X-ray Crystallography Unit, School of Physics, Universiti Sains Malaysia, 11800 USM, Penang, Malaysia; b Organic Chemistry Division, School of Advanced Sciences, VIT University, Vellore 632 014, India

## Abstract

The address of three of the authors in the paper by Shahani *et al.* [*Acta Cryst.* (2010), E**66**, o3020–o3021] is corrected.

## Abstract 2

Corrigendum to *Acta Cryst.* (2010), E**66**, o3020–o3021.

In the paper by Shahani *et al.* (2010) ▶, the address of the third, fourth and fifth authors is given incorrectly. The correct address is ‘Organic Chemistry Division, School of Advanced Sciences, VIT University, Vellore 632 014, India’, as given above.
